# New strategies for identifying and masking the bitter taste in traditional herbal medicines: The example of Huanglian Jiedu Decoction

**DOI:** 10.3389/fphar.2022.843821

**Published:** 2022-08-17

**Authors:** Xiumei Ke, Hongyan Ma, Junxuan Yang, Min Qiu, Jianwei Wang, Li Han, Dingkun Zhang

**Affiliations:** ^1^ College of Pharmacy, Chongqing Medical University, Chongqing, China; ^2^ State Key Laboratory Breeding Base of Systematic Research, Development and Utilization of Chinese Medicine Resources, School of Pharmacy, Chengdu University of Traditional Chinese Medicine, Chengdu, China

**Keywords:** mechanism, bitterness, bitterness suppression, Huanglian Jie-du Decoction, neotame, γ-CD

## Abstract

Suppressing the bitter taste of traditional Chinese medicine (TCM) largely has been a major clinical challenge due to complex and diverse metabolites and high dispersion of bitter metabolites in liquid preparations. In this work, we developed a novel strategy for recognizing bitter substances, hiding their bitter taste, and elucidated the mechanism of flavor masking in TCM. Huanglian Jie-Du Decoction (HLJDD) with an intense bitter taste was studied as a typical case. UHPLC-MS/MS was used to analyze the chemical components in HLJDD, whereas the bitter substances were identified by pharmacophores. Additionally, the screening results of the pharmacophores were further validated by using experimental assays. The mask formula of HLJDD was effectively screened under the condition of clear bitter substances. Subsequently, computational chemistry, molecular docking, and infrared characterization (IR) techniques were then used to explicate the mechanism of flavor masking. Consequently, neotame, γ-CD, and mPEG_2000_-PLLA_2000_ significantly reduced the bitterness of HLJDD. Specifically, mPEG_2000_-PLLA_2000_ increased the colloid proportion in the decoction system and minimized the distribution of bitter components in the real solution. Sweetener neotame suppressed the perception of bitter taste and inhibited bitter taste receptor activation to eventually reduce the bitter taste. The γ-CD included in the decoction bound the hydrophobic groups of the bitter metabolites in real solution and “packed” all or part of the bitter metabolites into the “cavity”. We established a novel approach for screening bitter substances in TCM by integrating virtual screening and experimental assays. Based on this strategy, the bitter taste masking of TCM was performed from three different aspects, namely, changing the drug phase state, component distribution, and interfering with bitter taste signal transduction. Collectively, the methods achieved a significant effect on bitter taste suppression and taste masking. Our findings will provide a novel strategy for masking the taste of TCM liquid preparation/decoction, which will in return help in improving the clinical efficacy of TCM.

## 1 Introduction

Traditional Chinese medicine (TCM) has an increasing impact on health and disease, with the recent prevention and treatment of COVID-19 serving as an example. Although “good medicine tastes bitter” is almost the basic feature of TCM, particularly for Chinese medicine decoction, the innate resistance of humans and mammals to bitter taste severely influences the compliance of patients (Amin et al., 2018) to TCM. This extremely affects the clinical efficacy of TCM (Zheng et al., 2018), specifically in children (Felton, 2018) and the elderly.

Currently, several methods for suppressing the bitter taste have been reported, including physical isolation of bitter substances, bitter taste receptor inhibitors (Masamoto et al., 2020 ; Andrews et al., 2021) which target the activation of bitter receptors, and inclusion of taste substances interfering with bitterness signal transduction (Wu et al., 2020). Although these methods have yielded certain effects in the application of modern medicine with single metabolites, they are ineffective in liquid preparations of TCM because of complex metabolites and prominent bitter taste. Therefore, the mechanism of improving the taste of TCM decoction without affecting the composition, efficacy, and side effects and also changing it from “good medicine tastes bitter” (Dang et al., 2020) to “good medicine tastes acceptable” requires research.

Huanglian Jie-Du Decoction (HLJDD) is a traditional Chinese herbal formula comprising *Coptis chinensis* Franch (Huanglian), *Scutellaria baicalensis* Georgi (Huangqin), *Phellodendron amurense* Rupr. (Huangbai), and *Gardenia jasminoides* J. Ellis (Zhizi) at a weight ratio of 3:2:2:3. This formula was first mentioned in the book “Wai-Tai-Mi-Yao” compiled by Wang Tao during the Tang dynasty and has been extensively used in clinical practice in China. Based on the TCM theory, HLJDD is the representative and basic prescription to clear heat, remove toxins, and eliminate the San Jiao fire toxin. Clinically, HLJDD is widely used as complementary and alternative medicine for treating sepsis, organ dysfunction syndrome, Alzheimer’s disease, pneumonia, meningitis, encephalitis (Chen et al., 2016), various cardiovascular diseases, diabetes mellitus, etc., Studies indicate that HLJDD could effectively relieve cardiac damage caused by metabolic disorders by improving inflammation-mediated insulin resistance (Chen et al., 2021). Moreover, HLJDD exhibits various pharmacological effects including hypolipidemic effect, anti-atherosclerosis effect, inhibitory effect on lipid peroxidation, anti-inflammatory effect, and cerebral protection effect (He et al., 2014).

**FIGURE 1:**
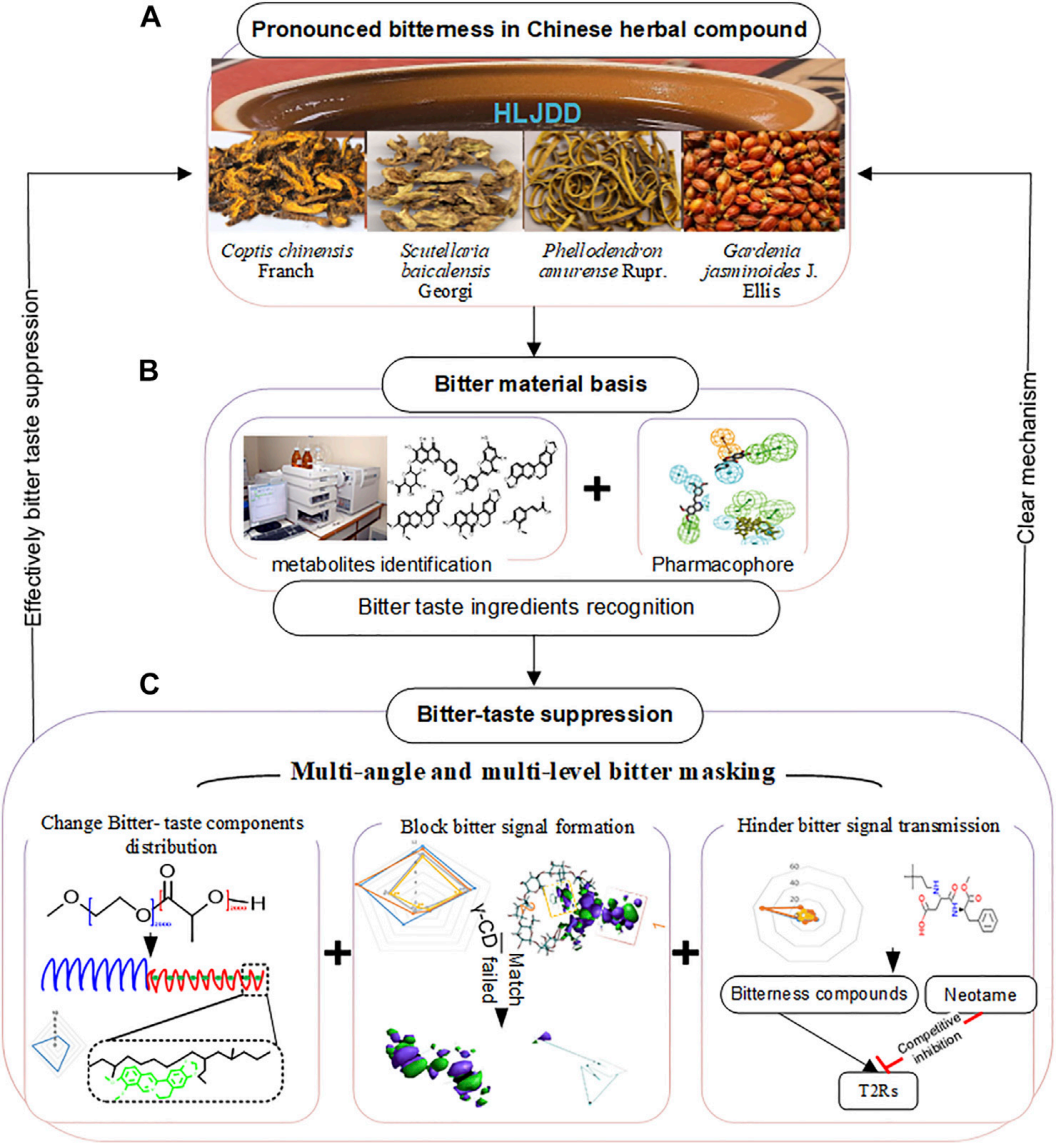
Graphic abstract. **(A)** HLJDD comprises Coptis chinensis Franch, Scutellaria baicalensis Georgi, Phellodendron amurense Rupr., and Gardenia jasminoides J. Ellis. It has a strong bitter taste. **(B)** UHPLC-MS/MS was used to identify the metabolites in HLJDD, and pharmacophores were used to recognize bitterness metabolites. **(C)** Multi-angle and multilevel bitter masking strategy with the amphiphilic block polymer, inclusion complex, and sweetener were applied to bitter-taste suppression of HLJDD. This new strategy achieved significant bitterness suppression and revealed the mechanism of bitter taste masking by combining the virtual analysis and experiment assays.

However, the compliance of adults and children to HLJDD is reduced by its strong bitter and slightly sour taste. This study investigated the mechanism of suppressing bitterness in HLJDD based on its components. Our results will provide novel insights for suppressing bitterness in TCM compounds Figure 1.

## 2 Materials and methods

### 2.1 Herbal materials and chemicals

*Coptis chinensis* Franch, *Scutellaria baicalensis* Georgi, *Phellodendron amurense* Rupr., and *Gardenia jasminoides* J. Ellis were all purchased from Sichuan Xinhehua TCM Decoction pieces Co., Ltd. (Sichuan, China) and were identified by lecturer Xiaofen Liu from the Pharmacognosy Department of Chengdu University of Traditional Chinese Medicine. Portions of the previously mentioned four herbs were deposited in the School of Pharmacy of Chengdu University of Traditional Chinese Medicine. Geniposide and chlorogenic acid standards (purity ≥95 %, HPLC) and phellodendron, epiberberine, baicalin, ferulic acid, berberine hydrochloride, wogonin, and obakunone standards (purity ≥98 %, HPLC) were obtained from Chengdu Keluoma Biological Technology Co., Ltd. (Sichuan, China). Thermo Fisher (America) supplied methanol, acetonitrile, and formic acid.

### 2.2 Preparation of Huanglian Jie-Du decoction and reference solution

#### 2.2.1 Preparation of Huanglian Jie-Du decoction

Coptidis Rhizoma, Scutellariae Radix, Phellodendri Chinensis Cortex, and Gardeniae Fructus decoction pieces were mixed at a ratio of 3:2:2:3 and decocted. The decoction pieces were soaked 10 times in pure water for 0.5 h, decocted twice for 1 h each time, filtered, and combined with a secondary filtrate. Exactly 10 g powdered HLJDD was boiled until the volume reached 100 ml, and 1 ml of the supernatant was filtered with a 0.22-μm filter.

#### 2.2.2 Preparation of the reference solution

Furthermore, 20 mg of chemicals, including geniposide, chlorogenic acid, phellodendron, epiberberine, baicalin, ferulic acid, berberine hydrochloride, wogonin, and obakunone, were accurately weighed and dissolved in 100 ml methanol.

### 2.3 Discovery of the compounds of bitter substances

#### 2.3.1 Combining virtual screening and experimental assays to identify bitter substances

##### 2.3.1.1 Analysis of Huanglian Jie-Du decoction substances using UHPLC-MS/MS conditions

Sample and reference chemicals were analyzed using a triple four-stage rod liquid mass spectrometer (UHPLC-30AD/AB SCIEX 4000, SHIMADZU, Japanese) and a high-resolution liquid-mass spectrometer (AB SCIEX TripleTOF™, AB SCIEX, American). Separation was performed on the C_18_ UHPLC column provided by Phenomenex (100 × 2.1 mm). The binary gradient elution system comprised (A) water (containing 0.1 % methanoic, v/v) and (B) acetonitrile (containing 0.1 % methanoic, v/v). Separation was achieved using the following gradient: 0 min, 5 % B; 1 min, 10 % B; 7 min, 85 % B; 11 min, 85 % B; 11.5 min, 10 % B; and 15 min, 10 % B. The flow rate was 0.3 ml/min, the column temperature was 30°C, and the injection volume was 2 μL. In addition, dynamic background subtraction (DBS) triggers the information association acquisition mode (IDA) which was used for scanning. Gas 1 and Gas 2 were both 55 Psi, the IS was 4500 V, the ionization temperature was 600°C, the curtain gas was 25 Psi, the collision voltage was 25 V, and the chamber injection voltage was 15 V. Data analysis was performed using AnalystTM1.6 software and MultiQuantTM3.0 software (American AB SCIEX). Eventually, the compounds with |ΔPPM | < 15.0 were derived (Xu et al., 2021).

##### 2.3.1.2 The pharmacophore models of bitter taste receptors (BTRs)

###### 2.3.1.2.1 The training set of the pharmacophore models

In humans, bitterness perception is mediated by 25 BTRs present in the oral cavity. Among them, Tas2r10, Tas2r14 (Nowak et al., 2018 ; Di Pizio et al., 2020), and Tas2r46 (Karaman et al., 2016), exhibited extraordinary wide agonist profiles, recognizing more than 50 % of natural bitter substances (Behrens and Meyerhof, 2018). Therefore, Tas2r10, Tas2r14, and Tas2r46 were selected to create pharmacophore models and identify bitter metabolites in HLJDD. The hip–hop algorithm in Discovery Studio 4.0 software (Accelrys, American) was used to construct a pharmacophore model based on Tas2r10, Tas2r14, and Tas2r46 ligands. Notably, the activity values of the 12 bitter compounds with BTRs were in accordance with those of previous studies (Meyerhof et al., 2010 ; Roland et al., 2011 ; Roland et al., 2013 ; Kuroda et al., 2016). Also, the activity values were expressed in terms of EC_50_ (concentration for 50 % of maximal effect, shown in Supplementary Table S1). Supplementary Figure S1 shows the structure of training test compounds.

###### 2.3.1.2.2 Construction of the pharmacophore models

To construct the pharmacophore models, five types of pharmacophore features were selected using Discovery Studio 4.0 software, including hydrogen bond acceptor (HBA), hydrophobic (H), hydrogen bond (HBD), hydrophobic aliphatic (HA), and ring aromatic (R). In constructing the model, the maximum number of conformations should be 255, and the choice of the model for the optimal is best. Moreover, the energy threshold should be 20 Kal/mol, a similar conformation energy threshold for each molecular homolog is 10, the number of the characteristic features of pharmacophore is 0–5, and the other parameter values are default (Han et al., 2020).

Tas2r10, Tas2r14, and Tas2r46 ligands were predicted by the BitterX (MDL.shsmu.edu.cn/bitterx/) website (Huang et al., 2016). Based on the principle of structural diversification and activity difference, epiberberine, coptisine, berberrubine, palmatine, eugenol, oroxylin, chlorogenic acid, and crocetin were selected as test sets to detect the activity of pharmacophores. Supplementary Table S2 presents the binding rates of compounds in the test set predicted by the Bitter X site with Tas2r10, Tas2r14, and Tas2r46, Supplementary Figure S2 shows the structure of the test set compounds.

###### 2.3.1.2.3 Identification of bitter metabolites

HLJDD compounds to be screened were imported into Discovery Studio 4.0 software, and we constructed a 3D database. The selected best pharmacophores were utilized to screen and identify the bitter compounds in HLJDD. Remarkably, default values of the software were used for parameters.

##### 2.3.1.3 Verification of virtual assay

###### 2.3.1.3.1 Method of taste evaluation

Quantitative description analysis was used to evaluate sample taste. A sweeter sensory index was used to define the sensory evaluation, and four sensory features and their references were screened as listed in Table 1. The evaluators were pre-trained based on the contents in Table 1. The sensory evaluation was performed twice.

**TABLE 1:** Sensory evaluation terms.

Term	Definition	Concentration (w/v) of reference-intensity	
		Sweet	Bitter
Taste at the beginning	Taste produced by sample solution after entering into the mouth for 30 s	Distilled water—0, 0.02 % neotame solution—10	Distilled water—0, 0.2 g/ml HLJDD decoction—10
Taste after swallowing	Taste after sample solution was spit for 30 s		

Distilled water was used for the preparation of sample solutions, and the solutions were placed in a constant temperature water bath at 40°C ± 1°C for 2 h before sensory evaluations. A 25-ml sample was provided each time with random coding and random tasting evaluation. Samples were spit into a stationary container after assessments, and 1-min intervals were allowed between each sample testing. Water and bun without sugar were provided between samples as palate cleansers to remove any residual flavors. The temperature of the sensory evaluation room was maintained at 25°C ± 2°C.

The sensory evaluation panel was selected by detecting the thresholds for sweet (0.3 % sucrose solution) and bitter (1.6 % quinine solution) from undergraduate and graduate students at Chengdu University of Traditional Chinese Medicine, which comprised eight females and seven males (18–25 years old).

###### 2.3.1.3.2 Assessment using a sensory evaluation panel

All metabolites in HLJDD could not be tasted due to workload and time limitations. Therefore, gardenoside (iridoid glycosides), phellodendron and berberine (alkaloids), baicalin (flavonoids), and chlorogenic acid (organic acids) were selected as representatives. Distilled water was used to prepare a 15 mg/ml sample solution of the abovementioned metabolites. The taste evaluation by the sensory evaluation panel was performed to verify the pharmacophore screened results. Pearson’s correlation analysis was conducted on the predicted value of the Bitter X website, pharmacophore matching value, and taste score of volunteers to establish the correlation between bitterness recognition results of the pharmacophore and the three parameters.

### 2.4 Formula of bitter taste suppression

The optimal bitter taste masking formula for HLJDD in previous studies is as follows: neotame: γ-CD: mPEG_2000_-PLLA_2000_: HLJDD = 0.028: 1.5: 0.15: 100 (m: m), as published by Ke et al., 2021. 1) 0.14 g neotame, 2) 7.5 g γ-CD, 3) 0.75 g mPEG_2000_-PLLA_2000_, 4) 0.14 g neotame +7.5 g γ-CD, 5) 0.14 g neotame + 0.75 g mPEG_2000_-PLLA_2000_, 6) 7.5 g γ-CD + 0.75 g mPEG_2000_-PLLA_2000_, and 7) 0.14 g neotame + 7.5 g γ-CD + 0.75 g mPEG_2000_-PLLA_2000_ was added into 500 ml 0.2 g/ml (content of slices) HLJDD. They were thoroughly stirred and vortexed to dissolve and suspend. Thereafter, the sensory evaluation panel evaluated their tastes.

### 2.5 The mechanism of suppressing bitter taste

#### 2.5.1 The taste masking mechanism of mPEG_2000_-PLLA_2000_

The infrared (IR) spectrum absorption of the samples was established using the tablet method, whereas the thin slices of gardenoside, chlorogenic acid, phellodendron, and epberberine were prepared using dry KBr (spectral purity) and dry KBr as blank. Fourier transform infrared spectroscopy required that the content of KBr in each slice was approximately 55 mg, whereas the sample content was about 1.0 mg.

#### 2.5.2 The taste masking mechanism of γ-CD

The planar structure of the compound was input into the Hyperchem 8.0 program in sequence. Next, it was regularized and then geometrically optimized according to the gradient value for three-dimensional imaging. The MM + molecular force field was used to optimize the molecular structure. Additionally, a semi-empirical approach Am1 was used to further optimize the molecular structure. Finally, QSAR parameters and thermodynamic parameters of various compounds were calculated for the optimized molecular structure.

The AMBER molecular force field algorithm was used to optimize the connected molecular structure, and no further calculation was made for the molecules that could not bind. Finally, QSAR parameters of well-combined molecules were calculated, and the changes of drug molecules before and after binding with cyclodextrin molecules were compared.

#### 2.5.3 The taste-correcting mechanism of neotame

The amino acid sequences of Tas2r10, Tas2r14, and Tas2r46 (Tas2r10 ID: NP_076410.1, Tas2r14 ID: NP_076411.1, and Tas2r46 ID: NP_795,368.2) were downloaded from the NCBI database. Homology modeling was performed on the I-TASSER server (Available online: https://zhanglab.ccmb.med.umich.edu/I-TASSER/) (Roy et al., 2010 ; Yang et al., 2015 ; Yang and Zhang, 2015). After selecting the best homologous models for Tas2r10, Tas2r14, and Tas2r46 bitter receptors, molecular docking was performed with baicalin (flavonoid), limonin (sesquiterpenlacton), geniposide (iridoid), epigberberine (alkaloid), and neotame.

SYBYL-X 2.0 software was used to perform durflex-dock. First, the protein was pretreated with the preparation protein structure, including extraction of ligand structure, repair of terminal residues, hydrogenation, energy minimization, and automatic ligand construction. Ligand structure preparation was then used to construct small molecules, including energy minimization and 2D and 3D structure of molecules. Eventually, the Surflex-Dock GeomX (SFXC) mode was used during docking to improve docking accuracy. Remarkably, values of docking parameters were set at default.

### 2.6 Ethical approval

Experimental protocols were approved by Chengdu University of Traditional Chinese Medicine Affiliated Hospital Medical Ethics Committee (No.2020SL-017).

### 2.7 Statistical analysis

Statistical analyses were conducted using IBM SPSS Statistics 21.0 (IBM Corp. Armonk, NY, United States), and data were presented as means ± SD. Differences among groups were determined by one-way analysis of variance (ANOVA followed by a post-hoc test) (LSD method). Data followed by *p* < 0.05 were considered statistically significant.

## 3 Results

### 3.1 Compounds of Huanglian Jie-Du decoction

**FIGURE 2:**
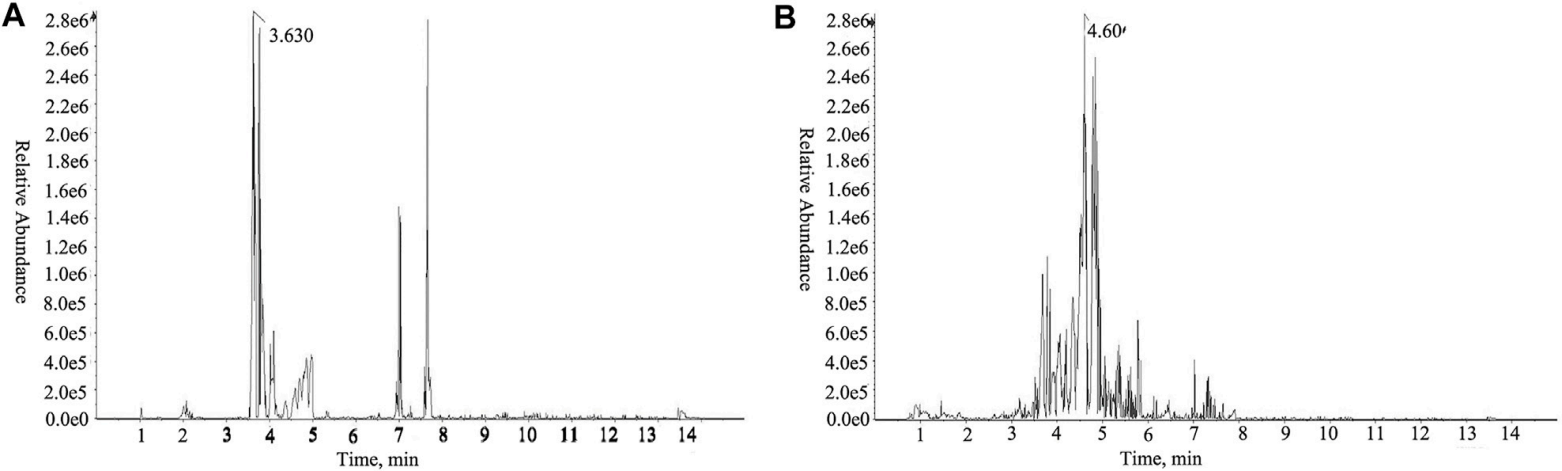
TIC detection diagram in the positive ion mode of UHPLC-Q-TOF-MS of HLJDD. **(A)** Mixed standard; **(B)** HLJDD.

UHPLC-Q-TOF-MS identified 35 compounds in HLJDD (Figure 2; Table 2). The compounds included five alkaloids (including berberine, hydrastine, and phellodendron), 15 flavonoids (including baicalin, wogonoside, and chrysin), five terpenoids (including genipin-1-β-glucoside), six organic acids (chlorogenic acid, ferulic acid, and benzoic acid), two phenols, one ketone, and one ester.

**TABLE 2:** Compounds in HLJDD.

Name	Molecular formula	Retention time/min	Molecular weight/Da	Adduct	Deviation/dppm
Berberine	C_20_H_18_NO_4_	4.90	336.12	M + H	−11.5
Obacunone	C_26_H_30_O_7_	7.67	454.20	M + H	−0.9
Obaculactone	C_26_H_30_O_8_	7.03	470.19	M + H	0.9
Oxyberberine	C_20_H_17_NO_5_	4.44	351.11	M + H	−0.4
Ferulic acid	C_10_H_10_O_4_	1.96	194.05	M + H	1.0
Coptisine	C_19_H_14_NO_4_	−14.6	320.09	M + H	4.63
Phellodendron	C_20_H_24_NO_4_	3.81	342.17	M + H	−14.2
Berberrubine	C_19_H_15_NO_4_	4.35	321.10	M + H	−0.3
Chlorogenic acid	C_16_H_18_O_9_	3.87	354.10	M + H	−1.4
Asperuloside	C_18_H_22_O_11_	2.02	414.12	M + H	0.6
Baicalin	C_21_H_18_O_11_	5.34	446.08	M + H	0.1
Baicalein	C_15_H_10_O_5_	6.49	270.05	M + H	0.3
Wogonoside	C_22_H_20_O_11_	5.82	460.10	M + H	−0.1
Skullcapflavone Ⅰ	C_17_H_14_O_6_	7.37	314.08	M + H	0.5
Skullcapflavone Ⅱ	C_19_H_18_O_8_	7.32	374.10	M + H	−0.3
Wogonin	C_16_H_12_O_5_	7.27	284.07	M + H	0.9
Norwogonin	C_15_H_10_O_5_	6.49	270.24	M + H	0.3
4-Heptenoic acid, 6-hydroxy	C_7_H_12_O_3_	3.92	144.17	M + H	−2.5
Eugenol	C_10_H_12_O_2_	2.26	164.08	M + H	0.7
Chrysin	C_15_H_10_O_4_	7.31	254.06	M + H	0.6
Dihydrobaicalein	C_15_H_12_O_5_	5.53	272.07	M + H	0.8
Dihydrooroxylin A	C_16_H_14_O_5_	7.48	286.08	M + H	−0.2
Crocetin	C_20_H_24_O_4_	4.71	328.17	M + H	0.3
Genipin-1-7-β-gentiobioside	C_23_H_34_O_15_	3.74	550.19	M + H	−1.3
Viscidulin Ⅱ	C_17_H_14_O_7_	6.49	330.07	M + H	−0.5
Viscidulin Ⅲ	C_17_H_14_O_8_	5.50	346.07	M + H	0.9
Baicalein-7-O-D-glucoside	C_21_H_20_O_10_	5.24	432.11	M + H	−0.7
Phthalic acid, diisohexylester	C_20_H_30_O_4_	7.45	334.21	M + H	−7.4
Benzoic acid	C_7_H_6_O_2_	3.12	122.04	M + H	0.2
Isophorone	C_9_H_14_O	4.76	138.10	M + H	0.6
4-Hydroxy-3-methoxy benzaldehyde	C_8_H_8_O_3_	1.87	152.05	M + H	0.5
(1S,3S,4S,5S)-1,3,4-trihydroxy-5-{[(2E)-3-(4-hydroxy-3-methoxyphenyl)prop-2-enoyl]oxy} cyclohexanecarboxylic acid	C_17_H_20_O_9_	4.43	368.11	M + H	−1.2
Myricitrin	C_21_H_20_O_12_	4.70	464.10	M + H	−1.2
Gardenolic acid B	C_30_H_46_O_5_	6.30	486.33	M + H	−1.2
Limonin	C_26_H_30_O_8_	7.03	471.19	M + H	0.9

We did not identify the high content of jatrorrhizine, palmatine, and geniposide in the herbs, potentially because of the high acidity and alkalinity of these components in decoction. Notably, jatrorrhizine and palmatine exhibit relatively strong alkalinity and certain solubility in water unlike other alkaloid components in HLJDD. At the same time, geniposide has strong acidity. During the hot and humid process of decocting, the neutralization reaction of acid–base components may be accompanied by desorption, dissolution, and diffusion of the herbs. This also justifies abundant yellow flocs during HLJDD decoction and storage.

### 3.2 Recognition of bitter taste compounds

#### 3.2.1 The pharmacophore models of bitter taste receptors

**FIGURE 3:**
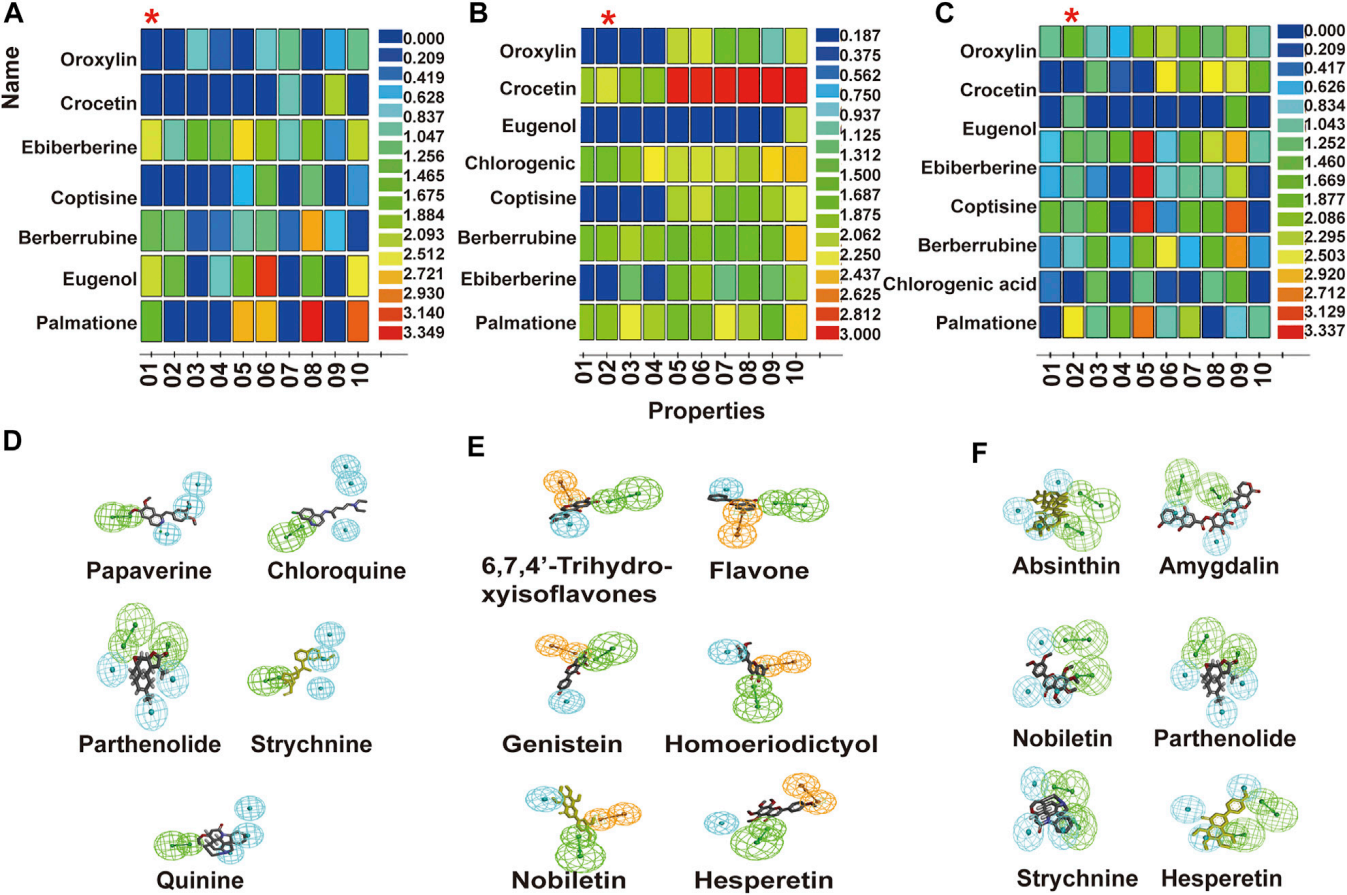
Bitter taste activity of pharmacophore predicted test set compounds and matching results of compounds in the training set and optimal pharmacophore. **(A,B,C)** show the top 10 pharmacophores of Tas2r10, Tas2r14, and Tas2r46. **(D,E,F)** show the matching results of compounds in the training set and optimal pharmacophore of Tas2r10, Tas2r14, and Tas2r46. The *X*-axis represents the pharmacophore (01–10 represents the order of the pharmacophore determined using software), and the *Y*-axis represents the compounds matching the pharmacophore. Blue indicates that the match is zero, and red shows that the matching activity is good.

Compounds in the test set were screened against pharmacophore 1, pharmacophore 2, and pharmacophore 2 from the top 10 pharmacophores of Tas2r10, Tas2r14, and Tas2r46, respectively (Figures 3A–C). The optimal pharmacophores of Tas2r10, Tas2r14, and Tas2r46 were as follows: Tas2r10 was described by four characteristics (HHHA), namely, three hydrophobic centers and one hydrogen receptor; Tas2r14 was described by three characteristics (RHA), namely, an aromatic ring center, a hydrophobic center, and a hydrogen receptor; and Tas2r46 was described by five characteristics (HHHAA), namely, two hydrogen receptors and three hydrophobic centers. Notably, all the pharmacophore characteristics were mapped to ligands. Figures 3D–F presents the matching results of the compound and the optimal pharmacophore.

#### 3.2.2 Bitter compounds in Huanglian Jie-Du decoction screened by the pharmacophores

The pharmacophores of Tas2r10, Tas2r14, and Tas2r46 screened 15, 16, and 15 compounds from HLJDD, respectively (Table 3). The results revealed that major bitter compounds in HLJDD included flavonoids in Scutellariae Radix, alkaloids and sesquiterpenlacton in Coptidis Rhizoma and Phellodendri Chinensis Cortex, and iridoids in Gardeniae Fructus.

**TABLE 3:** Bitter compounds in HLJDD screened using pharmacophores.

Tas2r14		Tas2r10		Tas2r46	
Compound	Match value	Compound	Match value	Compounds	Match value
Dihydrolignin A	2.8503	1.6686	0.0650		
Skullcapflavone Ⅱ	2.6838	2.6404	2.5801		
Phellodendron	2.6330	2.5021	2.2969		
Obacunone	2.6330	2.5021	2.2969		
Viscidulin Ⅱ	2.3705	2.4410	2.0450		
DIHP	2.2765	2.9818	3.7481		
Limonin	2.2337	1.2468	2.0251		
Baicalin	2.1538	3.3634	3.7969		
Viscidulin Ⅲ	1.6092	3.0228	3.1865		
Wogonoside	1.3285	2.7106	0.6229		
Wogonin	1.2365	1.9811	0.5815		
Scutellarin	1.1348	2.2650			
Baicalein -7-O-D-glucose	1.0192	2.5635			
Berberine	0.7152		1.5899		
Berberrubine	1.1534		2.1276		
Norwogonin	0.8530	Skullcapflavone Ⅰ	2.7558	Eugenol	1.4579
		Genipin-1-β gentiobioside	1.9157	Coptisine	1.1343

#### 3.2.3 Oral taste of bitter metabolites

Table 4 presents the results of oral taste of bitter metabolites, correlation analysis between them, and the predicted value by the Bitter X website as well as the matching value of the pharmacophores. Results showed a strong correlation between the predicted value and oral taste value (rs 0.708) and the predicted value and pharmacophore matching value (rs 0.686) of geniposide, baicalin, phellodendron, chlorogenic acid, and epiberberine. The pharmacophore matching value showed a certain correlation with the oral taste value (rs = 0.303), further verifying the effective identification of bitter taste components of the pharmacophore models in HLJDD. In addition, the matching value was linked to the bitterness of the ligand.

**TABLE 4:** Oral taste results (tasted bitter n = 15,‾x ± SD).

Compound	Matching value of pharmacophore	The predicted value of bitter X/%	Oral test value	Pearson’s test	
Geniposide	0	0	6.2 ± 2.1	rs	*p*
Phellodendron	4.930	238.1	15.8 ± 3.0		
Epiberberine	0	0	1.8 ± 0.5		
Baicalin	9.314	143.1	2.5 ± 0.6		
Chlorogenic Acid	0	60.6	0.5 ± 0.2		
	Pharmacophore vs. Bitter XPharmacophore vs. Oral test value Bitter X vs. Oral test value			0.708	0.115
				0.303	0.560
				0.686	0.133

PS: The matching value of the pharmacodynamics group and the predicted value of the Bitter X website in the table are obtained by adding the matching value/binding rate of each compound to Tas2r10, Tas2r14, and Tas2r46 (the detailed results of the matching value/binding rate of each component to the bitter taste receptor are shown in Supplementary Table S4).

### 3.3 The taste-masking effect of the taste mask formula

**FIGURE 4:**
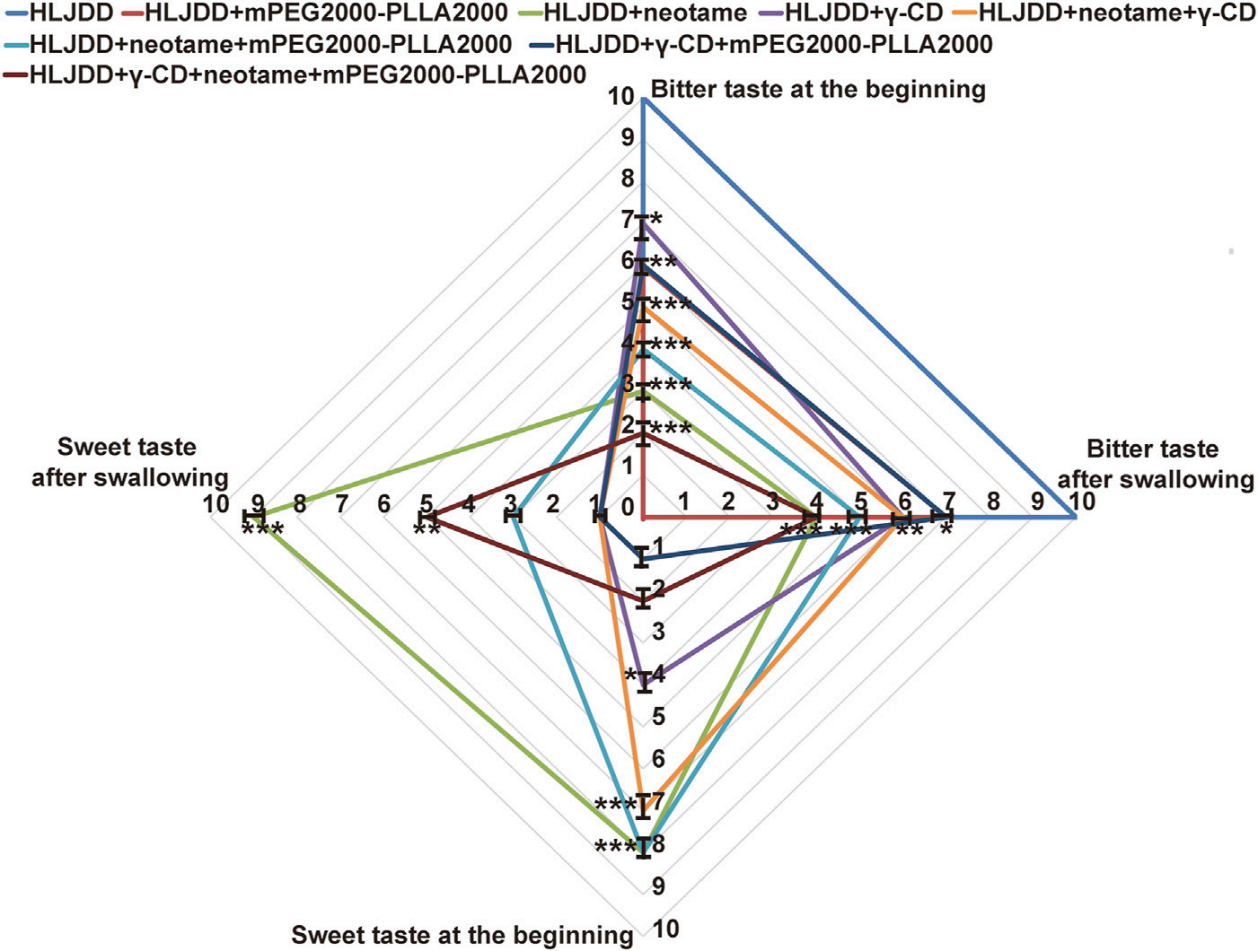
Taste-masking effect of taste mask formula (*n*=15, ‾x ± SD). Data shown are the mean± SD. *p<0.05, **p<0.01, and ***p<0.0001 versus HLJJD.

HLJDD had a strong bitter taste (Figure 4). The results revealed that the taste-masking agents [mPEG_2000_-PLLA_2000_ (0.15%), γ-CD (1.5%), and neotame (0.028%), m/m] moderately reduced the bitterness of HLJDD. Notably, 0.15% mPEG_2000_-PLLA_2000_ and 1.5% γ-CD partially decreased the intensity of HLJDD bitterness, whereas 1.5% γ-CD increased its sweetness. Additionally, 0.028% neotame decreased the bitterness and simultaneously increased the sweetness of HLJDD. Also, combining two of the three taste-masked agents significantly improved HLJDD texture with synergistic effects. γ-CD and mPEG_2000_-PLLA_2000_ exhibited the strongest synergistic effects in reducing the bitter taste in the HLJDD inlet. Neotame and mPEG_2000_-PLLA_2000_ demonstrated the strongest synergistic effects in reducing the bitterness in the duration of after-taste of HLJDD. γ-CD and neotame had the strongest synergistic effects in increasing the sweetness of HLJDD. The most important was that the γ-CD + neotame + mPEG_2000_-PLLA_2000_ formula achieved the strongest effect on taste correction. Neotame and mPEG_2000_-PLLA_2000_ had the largest and smallest contributions to the taste-masking effect of a corrigent for HLJDD, respectively.

In summary, a corrigent in the taste-masking formula improves the taste-masking effect, synergistically functioning with one another. Collectively, they minimized the bitterness and increased sweetness of HLJDD to achieve an effect of 1 + 1 + 1 > 3, and each is essential. In addition, the taste mask formula did not significantly influence the determination of primary chemical composition, the efficacy (*in vitro* antioxidant activity and inhibition of xylene induced ear swelling and anti-endotoxin induced hyperthermia in mice), safety (acute toxicity of mice), and potential side effects (intestinal flora) of TCM, in the case of HLJDD. (data not shown yet)

### 3.4 The taste masking mechanism

#### 3.4.1 IR characterization of the taste masking mechanism of mPEG_2000_-PLLA_2000_

**FIGURE 5:**
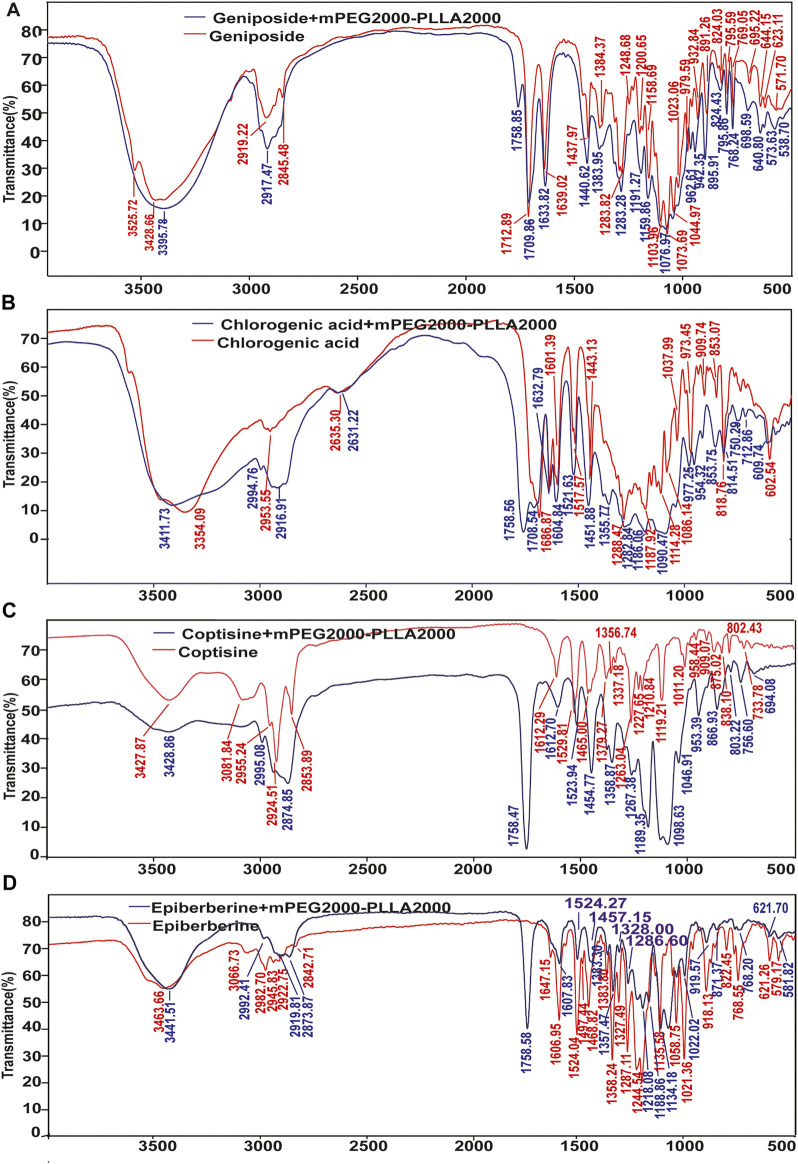
Results of IR analysis of the taste masking mechanism of mPEG2000-PLLA2000. **(A)** IR results of geniposide and geniposide + mPEG2000-PLLA2000, **(B)** IR results of chlorogenic acid and chlorogenic acid + mPEG2000-PLLA2000, **(C)** IR results of coptisine and coptisine + mPEG2000-PLLA2000, **(D)** IR results of epiberberine and epiberberine + mPEG2000-PLLA2000.

IR was used to explore the interaction between polymers and geniposide, chlorogenic acid, phellodendron, and epiberberine (Figure 5). Consequently, we found an additional absorption peak at 1758 cm^−1^ in the IR diagram of those compounds + mPEG_2000_-PLLA_2000_, suggesting that it was introduced by mPEG_2000_-PLLA_2000_. Notably, this peak was significant in epiberberine and phellodendron, which might be attributed to the interaction between -NH_2_ and mPEG_2000_-PLLA_2000_. Meanwhile, no fundamental change was noted in the chemical structure of these compounds, indicating no generation of novel compounds. The change was induced by an interaction of molecular forces in the complex between mPEG_2000_-PLLA_2000_ and these compounds.

The hydroxyl peaks at 3,525.72 cm^−1^ and 3,428.66 cm^−1^ shifted to low frequency in the geniposide + mPEG_2000_-PLLA_2000_. The peak widened and the peak number decreased, indicating the formation of hydrogen bonds between geniposide and mPEG_2000_-PLLA_2000_. Asymmetric stretching vibrations of C–H in the alkyl group changed from 2,919.22 cm^−1^ and 2,846.25 cm^−1^ to 2,917.47 cm^−1^, indicating that the hydroxyl groups in the molecule of geniposide might be influenced by the polar group of mPEG_2000_-PLLA_2000_. Furthermore, the blue shift of the absorption peak generally occurred after adding the mPEG_2000_-PLLA_2000_, and the group energy was lower and more stable (Figure 5A).

The absorption peaks of the phenolic hydroxyl group (3,444.5 cm^−1^ and 3,354.09 cm^−1^) in chlorogenic acid + mPEG_2000_-PLLA_2000_ were combined to the peak at 3,411.73 cm^−1^, hence widening the peak. Methylene vibration peak (2,953.55 cm^−1^) and other characteristic absorption peaks revealed a blue shift in IR spectra of chlorogenic acid + mPEG_2000_-PLLA_2000_, and no new peak appeared (Figure 5B). In addition, no strong hydroxyl vibration peak was noted at 3,400 cm^−1^; however, we noted a wide absorption peak at 3,400–2,500 cm^−1,^ and the vibration intensity was significantly changed. The absorption peak of the lowest spectral peaks of 1,500–500 cm^−1^ was blue-shifted. The results revealed that the hydroxyl group of phellodendron formed intermolecular forces with mPEG_2000_-PLLA_2000_ (Figure 5C). The absorption peak of hydroxyl 3,463.66 cm^−1^ in epiberberine + mPEG_2000_-PLLA_2000_ shifted to low frequency in the IR spectrum, whereas the peak number decreased with wider peak deformation. This indicates that an intermolecular force was formed between epiberberine and mPEG_2000_-PLLA_2000_ (Figure 5D).

#### 3.4.2 The taste masking mechanism of γ-cyclodextrin

##### 3.4.2.1 Optimization of the molecular structure of bitter taste components

Table 5 shows the calculation results of molecular 3D and thermodynamic parameters before self-assembly with γ-CD for bitter and sour components of HLJDD. The greater the dipole moment (D) and smaller the hydrophobicity parameter (*LogP*), the stronger the polarity and hydrophilicity will be. The results showed that γ-CD was the most hydrophilic and the most water-soluble compound compared with the bitter compounds.

**TABLE 5:** Molecular parameters calculated before self-assembly of γ-CD by bitter compounds in HLJDD.

Compound	MF	MW	G (TE/0 K)	S	D	SA	V	HE	Log *p*/300 K	G (TE/300 K)	Track
Berberine	C_20_H_18_NO_4_	336.36	−99360	0	0.89	757.40	940.23	-12.18	−2.91	−4,751	−7.59916
Obacunone	C_26_H_30_O_7_	454.51	−137932	0	2.13	603.06	1,106.07	182.77	1.94	−6,478	−9.55474
Limonin	C_26_H_30_O_8_	470.52	−138183	0	4.40	738.37	1,078.75	183.34	−0.63	−6,065	−9.68090
Ferulic Acid	C_10_H_10_O_4_	194.18	−62246	0	5.78	558.89	591.42	35.92	−0.63	−2,553	−8.90900
Coptisine	C_19_H_14_NO_4_	320.32	−95130	0	1.99	759.65	861.81	-15.18	2.40	−4,361	−7.89396
Berberrubine	C_19_H_15_NO_4_	321.33	−95765	0	2.23	734.92	887.99	-16.51	0.00	−4,464	−7.30709
Geniposide	C_17_H_24_O_10_	388.37	−98435	0	4.42	816.87	1,049.25	-16.15	4.74	−4,735	−8.14968
Chlorogenic Acid	C_16_H_18_O_9_	354.31	−119500	0	2.22	895.82	932.79	85.74	0.02	−4,510	−9.03248
Geniposidic Acid	C_16_H_22_O_10_	374.34	−128157	0	5.75	806.31	902.68	48.35	−2.02	−4,828	−9.48778
Crocetin	C_20_H_24_O_4_	328.41	−66094	0	6.45	514.86	578.22	-20.86	1.11	−2,512	−9.38015
Genipin-1-β-gentiobioside	C_23_H_34_O_15_	550.51	−189615	0	0.72	1,112.56	1,328.89	48.21	−3.20	−7,132	−9.61559
Eugenol	C_10_H_12_O_2_	164.20	−43322	0	1.78	891.75	919.14	-12.25	2.56	−952	−8.09896
Baicalin	C_21_H_18_O_11_	446.36	−149002	0	3.86	1,044.61	1,088.39	82.49	0.11	−5,488	−9.16880
Scutellarin	C_21_H_18_O_12_	462.37	−156393	0	3.32	1,110.53	1,099.94	138.03	−0.92	−5,538	−9.10321
Wogonoside	C_22_H_20_O_11_	460.39	−153226	0	1.52	986.94	1,124.91	98.54	5.64	-5,885	−9.14840
Wogonin	C_16_H_12_O_5_	284.26	−87957	0	5.20	706.61	768.06	33.24	1.50	-3,728	−9.17600
Skullcapflavone Ⅰ	C_17_H_14_O_6_	314.29	−98919	0	7.48	738.41	820.60	97.47	1.24	-4,092	−8.69704
Skullcapflavone Ⅱ	C_19_H_18_O_8_	374.35	−120867	0	4.04	784.29	969.96	34.06	0.74	-4,822	−8.84824
Norwogonin	C_15_H_10_O_5_	270.24	−84368	0	3.34	690.38	723.22	30.99	1.46	−3,457	−8.86331
Dihydrolignin A	C_16_H_14_O_5_	286.28	−88607	0	4.40	663.49	786.18	40.42	2.02	−3,835	−8.98775
Viscidulin Ⅱ	C_17_H_14_O_7_	330.29	−106317	0	7.83	792.24	852.50	31.40	0.96	−4,189	−8.94446
Viscidulin Ⅲ	C_17_H_14_O_8_	346.29	−113710	0	3.45	852.91	856.59	20.79	0.67	−4,290	−8.85996
Baicalein -7-O-D-glucoside	C_21_H_20_O_10_	432.38	−142244	0	3.50	948.61	1,049.71	32.24	0.00	−5,508	−9.09819
DIHP	C_20_H_30_O_4_	334.45	−98176	0	5.88	471.07	1,107.01	126.72	8.18	−5,335	−10.33996
γ-CD	C_48_H_80_O_40_	1,296.00	v462843	0	8.66	2,362.73	2,744.89	0.90	−9.73	−16218	−9.99965

PS: G, (Gibbs free energy); D, (dipole); S, (entropy); SA, (superficial area); TE, (total energy); HE, (hydration energy); MW, (molecular weight); MF, (molecular formula).

Most of the organic acids and bitter taste compounds selected using the pharmacophore had low hydration energy (HE) and strong hydrophobicity (*LogP*).

##### 3.4.2.2 Simulation results of self-assembly between bitter taste molecules and γ-cyclodextrin

Table 6; Supplementary Figure S3 shows the 3D and thermodynamic parameters of bitter and sour taste compounds after self-assembly with γ-CD and the differences before and after assembly. The sample entropy (SA) and Gibbs free energy (G) of guest molecules decreased after the host and guest molecules self-assembled into supramolecular molecules, indicating that the formation and stability of the system were easy. The dipole moment of most compounds increased significantly, suggesting that the self-assembled supramolecular compounds had higher hydrophilicity and lower hydrophobicity, which decreased binding affinity to the bitter receptor. Coptisine and eugenol could not self-assemble with γ-CD due to the failure of conformation matching. This may explain why γ-CD reduced the bitter and sour tastes of Scutellariae Radix and Gardeniae Fructus but not for Coptidis Rhizoma and Phellodendri Chinensis Cortex.

**TABLE 6:** Molecular parameters after self-assembly and the difference before and after self-assembly.

Name	SA	V	G	Log *p*	D	TE	ΔSA	ΔV	ΔG	ΔLog *p*	ΔD
Berberine	610.83	690.86	−550565	2.04	6.55	−550565	−146.57	−249.37	−451205	4.95	5.66
Obacunone	505.83	918.56	−593219	1.91	13.80	−593219	−97.23	−187.51	−455287	−0.03	11.67
Limonin	680.24	2008.77	−593622	−0.74	11.23	−593622	−58.13	930.02	−455439	−0.11	6.83
Ferulic Acid	483.59	453.56	−517046	1.73	6.65	−517046	−75.30	−137.86	−454800	2.36	0.86
Berberrubine	692.85	1988.60	−550551	0.11	8.46	−550551	−42.07	1,100.61	−454786	0.11	6.23
Geniposide	748.86	875.23	−553134	5.10	7.99	−553134	−68.01	−174.02	−454699	0.36	3.57
Chlorogenic Acid	810.20	2,536.98	−574287	1.75	7.05	−574287	−85.62	1,604.19	−454787	1.73	4.83
Geniposidic acid	704.61	796.03	−582951	−1.91	7.41	−582951	−101.70	−106.65	−454794	0.11	1.66
Crocetin	432.42	416.68	−520944	5.78	11.16	−520944	−82.44	−161.54	−454850	4.67	4.71
Genipin-1-β-gentiobioside	1,003.31	582.58	−644480	−2.85	4.12	−644480	−109.25	−746.31	−454865	0.35	3.40
Baicalin	908.91	857.78	−603775	0.22	5.76	−603775	−135.70	−230.61	−554773	0.11	1.90
Scutellarin	1,062.43	4,392.42	−611223	4.91	8.19	−611223	−48.10	3,292.48	−454830	5.83	4.88
Wogonoside	882.97	901.93	−607274	2.67	3.57	−607274	−103.97	−222.98	−454048	−2.97	2.05
Wogonin	567.36	2,110.94	−542720	1.61	10.48	−542720	−139.25	1,342.88	−454763	0.11	5.28
Skullcapflavone Ⅰ	647.65	2009.56	−553693	1.35	12.15	−553693	−90.76	1,188.96	−454774	0.11	4.67
Skullcapflavone Ⅱ	664.39	4,069.28	−575627	3.02	11.36	−575627	−119.90	3,099.32	−454760	2.28	7.32
Norwogonin	586.17	1756.06	−539202	1.58	8.98	−539202	−104.21	1,032.84	−454834	0.12	5.65
Dihydrolignin A	561.07	2,101.47	−543375	2.13	11.89	−543375	−102.42	1,315.29	−454768	0.11	7.49
Viscidulin Ⅱ	680.28	1,575.95	−561155	0.80	6.71	−561155	−111.96	723.45	−454838	−0.16	−1.12
Viscidulin Ⅲ	556.24	622.74	−543459	4.05	0.00	−543459	−296.67	−233.85	−429749	3.38	−3.45
Baicalein -7-O-D-glucoside	793.92	1,678.38	−597099	0.11	5.00	−597099	−154.69	628.67	−454855	0.11	1.50
DIHP	435.68	1,406.23	−560351	4.93	12.59	−560351	−35.39	299.22	−462175	−3.25	6.72

It should be noted that γ-CD binds to certain groups to sequester the bitter or sour component molecules (some or all) into the internal cavity, thereby alleviating the bitter taste of the molecules. Molecular simulation results indicated that organic acids and bitter components had low hydration energy (HE) and strong hydrophobicity (large *logP* value), which resulted in a weak binding force between these compounds and water molecules. The absence of γ-CD in the system favored entry of the bitter compounds to the active binding site (hydrophobic cavity) of the bitter taste receptor through hydrophobic interaction. In this way, activation of the bitter taste receptor was prevented. However, γ-CD creates a hydrophobic effect that pushes all or part of the compound (the hydrophobic group) into the γ-CD’s hydrophobic cavity, where it binds to its hydrophobic group to mask the taste. Moreover, after self-assembly with γ-CD, the hydrophilicity of bitter compounds increases, whereas hydrophobicity weakens, which reduces the affinity of these compounds to BTRs. Previous studies have reported that this is another mechanism through which γ-CD suppresses bitterness and masks the bitter taste (Allen et al., 2013 ; Di Pizio et al., 2019).

Although γ-CD has a good masking effect on the bitterness and sourness of Gardeniae Fructus decoction, it is not effective for drugs with strong bitterness such as Coptidis Rhizoma and Phellodendri Chinensis Cortex. This phenomenon may be attributed to two reasons. First, under the conditions, the host molecule (γ-CD) has a large molecular weight (M_w_ = 1,296) and low concentration, and the number of guest molecules (bitter and sour components) exceeds that of the host molecules. For this reason, γ-CD cannot bind all guest molecules in Coptidis Rhizoma and Phellodendri Chinensis Cortex. Second, the host–guest recognition depends on conformational matching. When strong bitter components such as coptisine and eugenol are close to γ-CD, a strong hydrophobic interaction is created which prevents the host–guest molecules from combining and undergoing self-assembly. Collectively, these factors suggest that γ-CD alone cannot mask the taste effectively, especially for liquid preparations of TCM with strong bitterness, complex components, diverse structures, and large molecular weight spans.

### 3.5 The taste-correction mechanism of neotame

**FIGURE 6:**
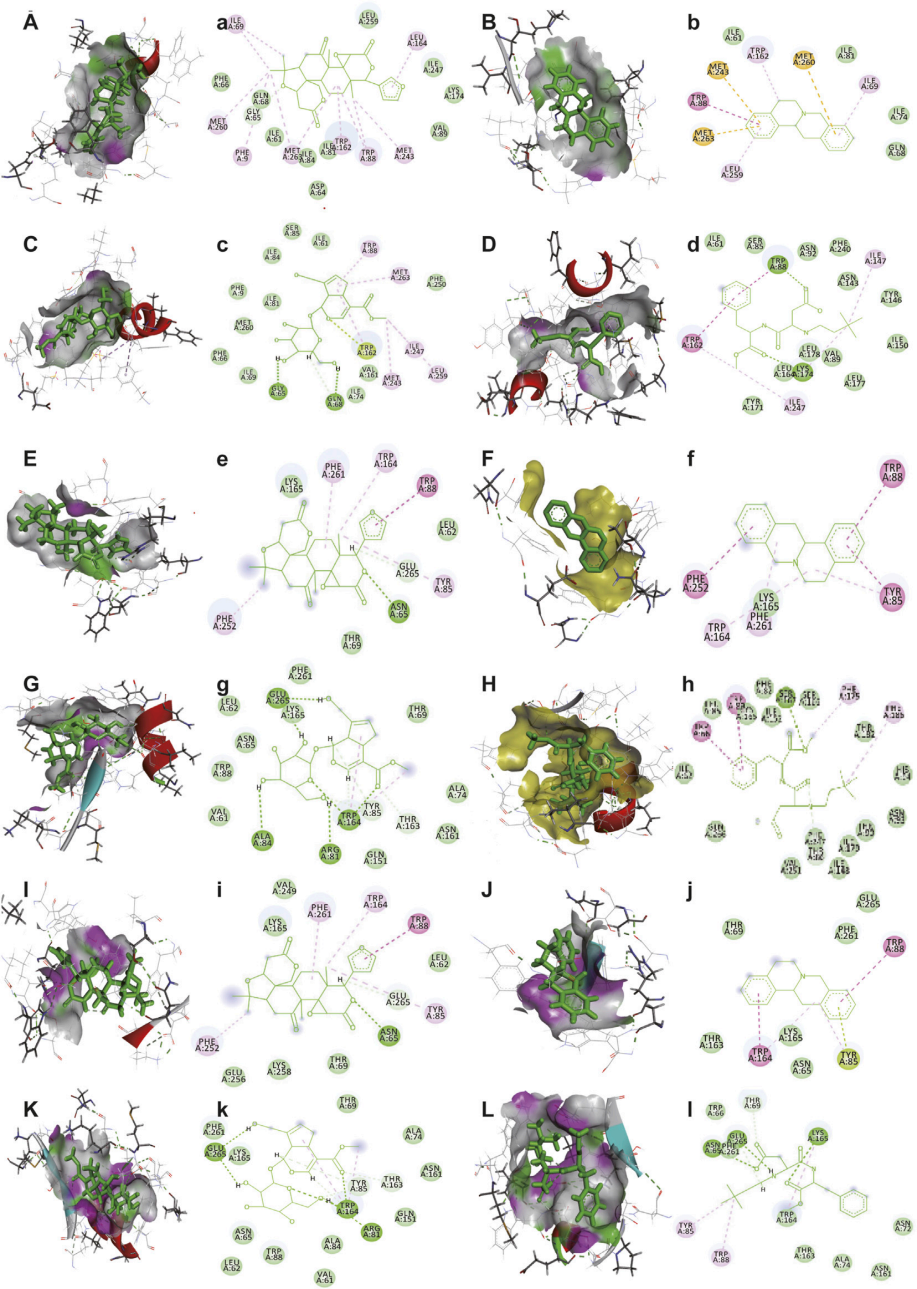
Interaction diagrams of bitter receptors with limonin, epiberberine, geniposide, and neotame. **(A–L)** Active pockets of limonin, epiberberine, geniposide, and neotame in Tas2r10, Tas2r14, and Tas2r46, respectively. (a–l) 2D diagram of receptor (Tas2r10, Tas2r14, and Tas2r46)-ligand (limonin, epiberberine, geniposide, and neotame), respectively. **(A,a)** Tas2r10 with limonin, **(B,b)** Tas2r10 with epiberberine, **(C,c)** Tas2r10 with geniposide, **(D,d)** Tas2r10 with neotame, **(E,e)** Tas2r14 with limonin, **(F,f)** Tas2r14 with epiberberine, **(G,g)** Tas2r14 with geniposide, **(H,h)** Tas2r14 with neotame, **(I,i)** Tas2r46 with limonin, **(J,j)** Tas2r46 with epiberberine, **(K,k)** Tas2r46 with geniposide, and **(L,l)** Tas2r46 with neotame. Light green indicates van der Waals interaction. Green indicates hydrogen bond interaction. Rose-red indicates pi–pi interaction. Sulfur yellow indicates pi–sulfur interaction.

The bitter receptors of Tas2r10, Tas2r14, and Tas2r46 models were built (Supplementary Figure S5), and then molecular docking was performed. Figure 6 and Table 7 show the molecular docking results of bitter receptors Tas2r10, Tas2r14, and Tas2r46 with limonin, geniposide, epigberberine, and neotame. The highest total score and CScore were selected from 20 docking conformations following software simulation (see Supplementary Figure S4 for the best conformation of compounds interacting with the receptor). With regard to Tas2r10 and Tas2r14 receptors, the affinity of neotame was significantly stronger than that of limonin, geniposide, and epigberberine. On the other hand, the affinity of neotame for Tas2r46 was significantly higher than that of limonin and berberine, but not different from that of geniposide. Interestingly, neotame had a strong binding force with Tas2r10, Tas2r14, and Tas2r46 (significantly higher than that of limonin, geniposide, and berberine) and reduced the bitter taste, suggesting that neotame can inhibit these bitter receptors.

**TABLE 7:** Docking results of bitter receptors and compounds.

Receptor	Grading type	Geniposide	Limonin	Epiberberine	Neotame
Tas2r10	Total score	6.64	5.68	3.34	7.94
	CScore	3.00	4.00	5.00	5.00
Tas2r14	Total score	7.41	5.43	3.71	8.73
	CScore	4.00	5.00	5.00	5.00
Tas2r46	Total score	8.38	5.79	4.53	8.03
	CScore	5.00	4.00	4.00	4.00

## 4 Discussion

Currently, the bitter taste of some medicines is a common cause of reduced patient compliance to TCM prescriptions, especially in children and the elderly (Li et al., 2020). Moreover, it is much more difficult to mask the bitter taste in TCM decoction than in Western medicine due to the complex components of TCM. This study established a novel strategy for detecting bitter substances and masking bitterness and elucidated the taste masking mechanism. HPLC/MS and pharmacophore were used to identify bitter substances, and the effective prescription of bitter suppression was screened. Finally, the taste masking mechanism was explored through IR, computational chemistry, and molecular docking.

The distribution of bitter metabolites in the complex system of TCM decoction (composed of a real solution, colloid, and suspension) is complex. Some metabolites only exist in real solution, colloid, or suspension, whereas some are distributed in two phases (such as real solution and colloid) or all three phases. This distribution is dynamic and changes over time with fluctuations in external conditions, including temperature, pH, and other conditions such as the addition of amphiphilic polymers. The bitter components under different phases can stimulate BTRs, with components in the real solution inducing the strongest stimulation effects.

The equilibrium between phase and bitter composition in TCM decoction was broken by the addition of the bitter taste receptor inhibitor. First, the amphiphilic block polymer (for example mPEG_2000_-PLLA_2000_) rapidly formed micelles in the sample and which entrapped the bitter components, increased the colloid proportion in the system, and decreased the bitter composition in the real solution. Based on the three-point contact theory, the bitter functional groups in bitter compounds are hydrophobic. The γ-CD is a barrel-like structure with an inner hydrophobic domain and an outer hydrophilic part. In the present study, we found that the binding site of γ-CD was fixed, whereas the locations of bitter metabolite-binding sites were in the terminal part, middle portion, or entire molecule (Supplementary Figure S3). This suggests that the terminal part, middle portion, or entire molecules of bitter metabolites might bind γ-CD during the self-assembly of host and guest molecules into supramolecular molecules. This caused the binding of γ-CD to the hydrophobic groups of the bitter metabolites and “packaging” all or part of the bitter metabolites into a barrel. From the perspective of medicines, the bitterness suppression methods described above protect bitter metabolites layer by layer, thereby preventing contact between bitter metabolites and receptors and the generation of bitter signals. From a human perspective, taste correction agents (such as sweetener neotame) inhibit BTR activation and prevent the transmission of bitter taste signals.

At present, a strategy for suppressing bitterness was established. The strategy involved changing the phase state of TCM decoction, distributing bitter metabolites in real solution and the electronic cloud state, and modifying the initiation and processing of bitter signals, thereby achieving multi-angle and multilevel bitter masking. Moreover, the taste-masking strategy comprehensively achieved masking and interference of bitter receptor activation. In addition, the bitter receptors activated by the remaining bitter metabolites were inhibited to achieve complete masking of the bitter taste.

Mammals perceive five basic gustatory sensations: sweet, sour, bitter, salty, and umami (Ke et al., 2020). These taste sensations interfere with each other. A previous study found that umami can suppress bitterness and enhance sweetness and saltiness (Keast and Breslin, 2002). Results of this study showed that neotame, an artificial sweetener, effectively masked the bitter taste of HLJDD. Molecular docking results also showed that neotame inhibited “broad-spectrum” bitter receptors (Tas2r10, Tas2r14, and Tas2r46) (Born et al., 2013 ; Fierro et al., 2019) and suppressed the bitter taste. Therefore, neotame interferes with upstream (source) and downstream (process) signals by preventing activation and transmission of bitter signals, thereby achieving flavor correction.

Of note, HLJJD is an ideal representative of the bitter-tasting TCM prescriptions. Moreover, bitter-tasting Chinese herbs such as Coptidis Rhizoma, Phellodendri Chinensis Cortex, and Gardeniae Fructus in HLJJD are widely used in clinical practice. Furthermore, the major categories of bitter compounds were alkaloids, flavones, iridoids, and glycosides, which are highly representative of bitter tasting TCM. The taste masking formula can effectively mask the bitterness of HLJJD and other bitter Chinese medicines. The applicability of the taste mask formula in TCM was tested on Sanhua decoction, Banxia Xiexin decoction, Danggui Liuhuang decoction, Huanglian decoction, Qingwei san, Andrographis paniculata, and several Chinese medicines. The results showed that the formula successfully improved the taste of these medicines. Considering that these prescriptions constitute a large proportion of bitter Chinese medicines, the strategy established in this study can be applied to multiple TCM medicines.

In summary, the bitter components of HLJDD were identified, and a taste-masking strategy for the bitter taste was developed. The results provide novel ideas for flavor correction in TCM decoctions. Taste improvement for TCM medicines can increase the clinical efficacy and compliance among children and elderly patients. However, given the complexity of the study drugs, we did not explore the interactions among bitter components and with bitter receptors. Moreover, the cross-interference between bitter components and receptors was not elucidated.

## Data Availability

The original contributions presented in the study are included in the article/Supplementary Material; further inquiries can be directed to the corresponding authors.

## Abbreviations

BTRs, bitter taste receptors; D, dipole; G, Gibbs free energy; H, hydrophobic; HA, hydrophobic aliphatic; HE, hydration energy; HBA, hydrogen bond acceptor; HBD, hydrogen bond; HLJDD, Huanglian Jie-Du Decoction; MF, molecular formula; MW, molecular weight; R, ring aromatic; S, sample entropy; SA, superficial area; TE, total energy; TCM, traditional Chinese medicine; γ-CD, γ-cyclodextrin.
