# Supplementary material for: New strategies for identifying and masking the bitter taste in traditional herbal medicines: The example of Huanglian Jiedu Decoction
Source: Front Pharmacol. 2022 Aug 17;13:843821. doi: 10.3389/fphar.2022.843821 (PMC9431955; doi:10.3389/fphar.2022.843821)
Supplement: Supplementary file 1 [file DataSheet1.docx]

**Supplementary material**

Supplementary material in this article is as following:

**1 The structure of training test compounds**


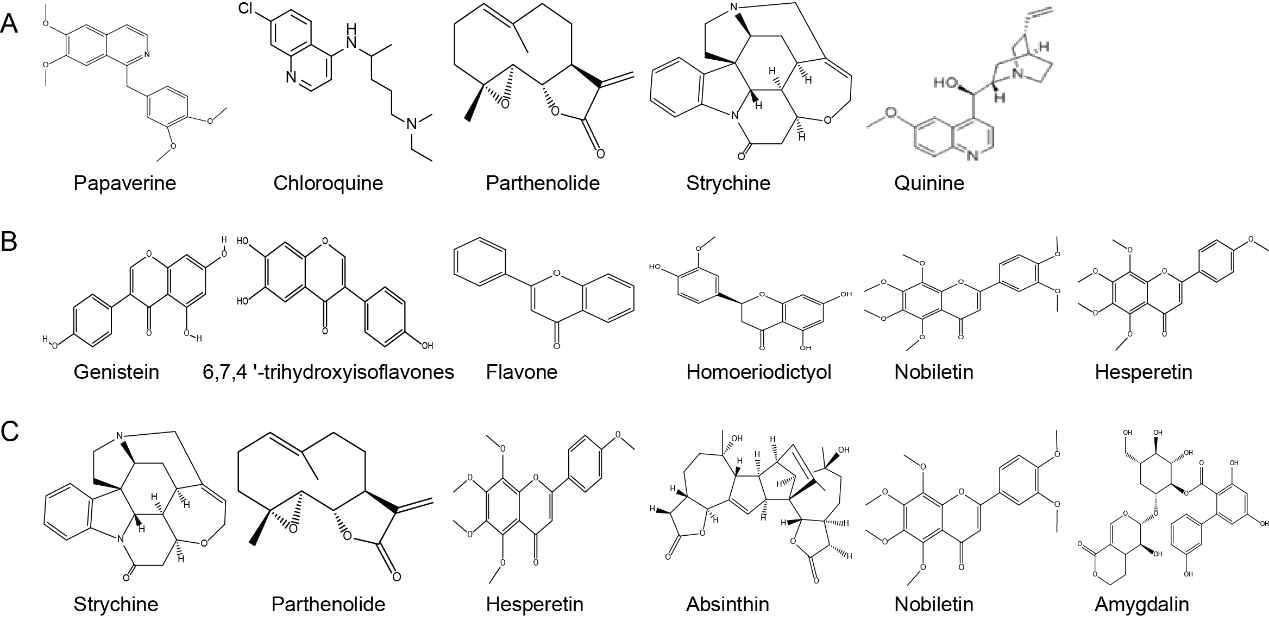


Fig.S.1 The structure of compounds in training set, A shows the structure of compounds in trainning set of Tas2r10, B shows the structure of compounds in training set of Tas2r14, C shows the structure of compounds in training set of Tas2r46.

**2 The structure of compounds in the test set**


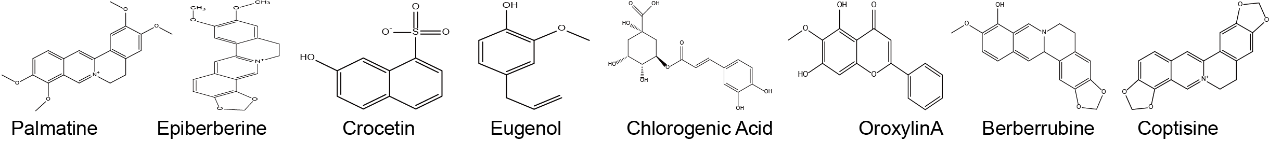


Fig.S.2 The structure of compounds in the test set

**3 The optimized molecular structure and binding sites**


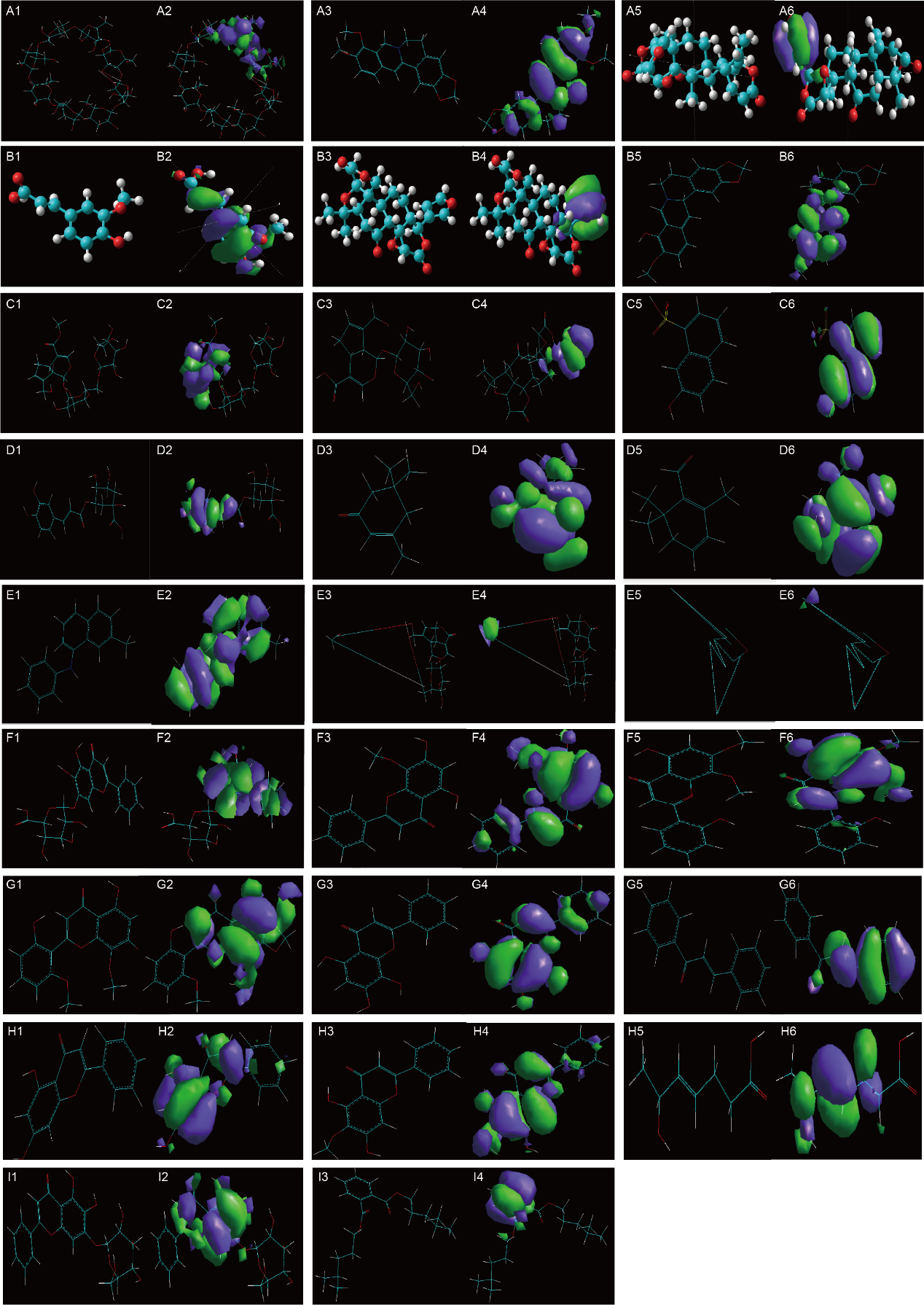


Fig.S.3 The optimized molecular structure and binding sites. A1 shows structure of γ-CD. A2 shows binding site of γ-CD. A3 shows structure of berberine. A4 shows binding site of berberine. A5 shows structure of obacunone. A6 shows binding site of obacunone. B1 shows structure of limonin. B2 shows binding site of limonin. B3 shows structure of ferulic acid. A4 shows binding site of ferulic acid. B5 shows structure of coptisine. B6 shows binding site of coptisine. C1 shows structure of berberrubine. C2 shows binding site of berberrubine. C3 shows structure of geniposide. C4 shows binding site of geniposide. C5 shows structure of chlorogenic acid. C6 shows binding site of chlorogenic acid. D1 shows structure of geniposidic acid. D2 shows binding site of geniposidic acid. D3 shows structure of crocetin. D4 shows binding site of crocetin. D5 shows structure of genipin-1-β-gentiobioside. D6 shows binding site of genipin-1-β-gentiobioside. E1 shows structure of eugenol. E2 shows binding site of eugenol. E3 shows structure of baicalin. E4 shows binding site of baicalin. E5 shows structure of scutellarin. E6 shows binding site of scutellarin. F1 shows structure of wogonoside. F2 shows binding site of wogonoside. F3 shows structure of wogonin. F4 shows binding site of wogonin. F5 shows structure of skullcapflavone Ⅰ. F6 shows binding site of skullcapflavone Ⅰ. G1 shows structure of skullcapflavone Ⅱ. G2 shows binding site of skullcapflavone Ⅱ. G3 shows structure of norwogonin. G4 shows binding site of norwogonin. G5 shows structure of dihydrolignin A. G6 shows binding site of dihydrolignin A. H1 shows structure of viscidulin Ⅱ. H2 shows binding site of viscidulin Ⅱ. H3 shows structure of viscidulin Ⅲ norwogonin. H4 shows binding site of viscidulin Ⅲ. H5 shows structure of dihydrolignin A. H6 shows binding site of dihydrolignin A. I1 shows structure of baicalein -7-O-D-glucoside. I2 shows structure of baicalein -7-O-D-glucoside. I3 shows structure of DIHP. I4 shows structure of DIHP.

**4 3D picture of the best conformation of compound**


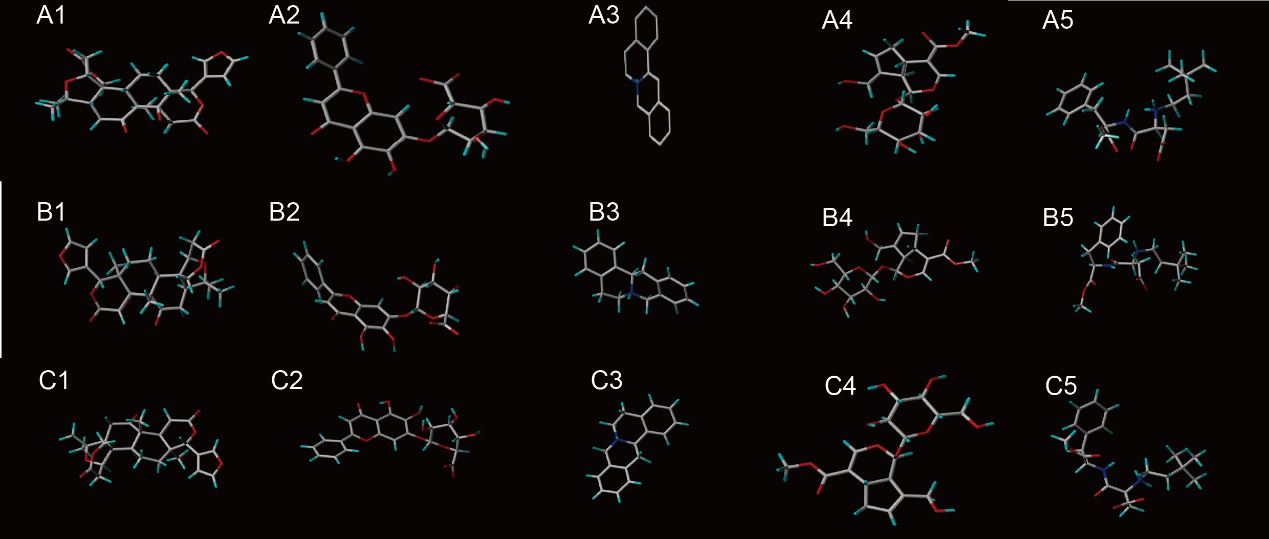


Fig.S.4 3D picture of the best conformation of compound. A shows the best conformation of compound when binding with Tas2r10, B shows the best conformation of compound when binding with Tas2r14, C shows the best conformation of compound when binding with Tas2r46. 1 is limonin, 2 is baicalin, 3 is epiberberine, 4 is geniposide, 5 is neotame.

**5 EC_50_ of compounds and bitter receptor in training set**

Table S.1 EC_50_ of compounds and bitter receptor in training set

| Bitter taste receptor | EC_50_ (μM) | | | | | |
| --- | --- | --- | --- | --- | --- | --- |
| Tas2r10 | Chloroquine | Parthenolide | Strychnine | Papaverine | Quinine |  |
|  | 10000.00 | 30.00 | 21.80 | 10.00 | 10.00 |  |
| Tas2r14 | 6,7,4 '-trihydroxyisoflavones | Homoeriodictyol | Genistein | Flavone | Nobiletin | Hesperetin |
|  | 378.00 | 63.90 | 28.90 | 20.50 | 2.41 | 0.33 |
| Tas2r46 | Parthenolide | Absinthin | Hesperetin | Nobiletin | Amygdalin | Strychnine |
|  | 1.00 | 9.90 | 4.53 | 3.81 | 65.00 | 0.43 |

**6 The binding properties of compounds to bitter taste receptors from BitterX website**

Table S.2 The binding properties of compounds to bitter taste receptors from BitterX website

| BTRs | Epiberberine | Crocetin | Coptisine | Berberrubine | Eugenol | OroxylinA | chlorogenic acid | Palmatine |
| --- | --- | --- | --- | --- | --- | --- | --- | --- |
| Tas2r10 | 0 | 0 | 0 | 0 | 66.71 | 57.97 | 0 | 76.38 |
| Tas2r14 | 0 | 0 | 0 | 0 | 65.66 | 76.11 | 60.61 | 70.94 |
| Tas2r46 | 0 | 0 | 0 | 0 | 51.86 | 62.85 | 0 | 75.91 |

**7 Pharmacophore modeling results**

The ligands of bitter receptors Tas2r10 and Tas2r46 both take hydrophobic center (H) and hydrogen bond receptor (A) as the basic pharmacophore elements, and the ligands of Tas2r14 take aromatic ring center (R) and A as the basic pharmacophore element. Among them, H and A are the common features of the three bitter receptor pharmacophores, and correspond to the core of the training set compound of the three receptors.

Table S.3 Pharmacophore modeling results

| BTRs | Pharmacophore | Features | Rank | Direct Hit | Partial Hit | Max Fit |
| --- | --- | --- | --- | --- | --- | --- |
| Tas2r10 | 01 | HHHA | 34.627 | 1111 | 10000 | 4 |
|  | 02 | HHAA | 34.434 | 11110 | 1 | 4 |
|  | 03 | HHAA | 34.319 | 11110 | 1 | 4 |
|  | 04 | HHAA | 34.199 | 11110 | 1 | 4 |
|  | 05 | HHHA | 33.833 | 1111 | 10000 | 4 |
|  | 06 | HHHA | 33.712 | 1111 | 10000 | 4 |
|  | 07 | HHAA | 33.366 | 11110 | 1 | 4 |
|  | 08 | HHHA | 33.348 | 1111 | 10000 | 4 |
|  | 09 | HHAA | 33.275 | 11110 | 1 | 4 |
|  | 10 | HHHA | 33.015 | 1111 | 10000 | 4 |
| Tas2r14 | 01 | RHA | 37.932 | 111111 | 0 | 3 |
|  | 02 | RHA | 37.790 | 111111 | 0 | 3 |
|  | 03 | RHA | 34.604 | 111111 | 0 | 3 |
|  | 04 | RHA | 34.604 | 111111 | 0 | 3 |
|  | 05 | RRA | 33.592 | 111111 | 0 | 3 |
|  | 06 | RRA | 33.592 | 111111 | 0 | 3 |
|  | 07 | RRA | 33.592 | 111111 | 0 | 3 |
|  | 08 | RRA | 33.312 | 111111 | 0 | 3 |
|  | 09 | RRA | 33.298 | 111111 | 0 | 3 |
|  | 10 | RHA | 31.192 | 111111 | 0 | 3 |
| Tas2r46 | 01 | HHHAA | 54.491 | 111101 | 10 | 5 |
|  | 02 | HHHAA | 51.501 | 111101 | 10 | 5 |
|  | 03 | HHAA | 50.377 | 111111 | 0 | 4 |
|  | 04 | HHAA | 50.320 | 111111 | 0 | 4 |
|  | 05 | HHHAA | 50.019 | 111101 | 10 | 5 |
|  | 06 | HHAA | 47.784 | 111111 | 0 | 4 |
|  | 07 | HHAA | 47.784 | 111111 | 0 | 4 |
|  | 08 | HHAA | 47.310 | 111101 | 10 | 4 |
|  | 09 | HHAA | 46.946 | 111111 | 0 | 4 |
|  | 10 | HHAA | 46.250 | 111111 | 0 | 4 |

**8 The matching value/binding rate of each compound and bitter taste receptor**

Table S.4 The matching value/binding rate of each compound and bitter taste receptor

| BTR | | Geniposide | Phellodendrine | Epiberberine | Baicalin | Chlorogenic Acid |
| --- | --- | --- | --- | --- | --- | --- |
| Tas2r10 | Pharmacophore matching values | 0 | 2.5021 | 0 | 3.3634 | 0 |
|  | Predicted value of Bitter X /% | 0 | 84.03 | 0 | 0 | 0 |
| Tas2r14 | Pharmacophore matching values | 0 | 2.6330 | 0 | 2.1538 | 0 |
|  | Predicted value of Bitter X /% | 0 | 71.91 | 0 | 72.91 | 60.61 |
| Tas2r46 | Pharmacophore matching values | 0 | 2.2969 | 0 | 3.7969 | 0 |
|  | Predicted value of Bitter X /% | 0 | 82.16 | 0 | 70.18 | 0 |

**9 Results of homology modeling**

Fig. S 5 shows the evaluation results of the bitter receptors of Tas2r10, Tas2r14, and Tas2r46 models. Notably, RMSDa is the RMS deviation of residues in sequence alignment (the lower the better); and Cov. is the residual coverage compared with the template (the higher the better). In this study, RMSDa < 2.0 and cov. > 0.9, they were all in a high-level range, suggesting good models. The TM-score of each model was > 0.8, indicating that the modeling results were reliable. A TM-score > 0.5 indicated that the structure of the model was similar to that of the natural protein.


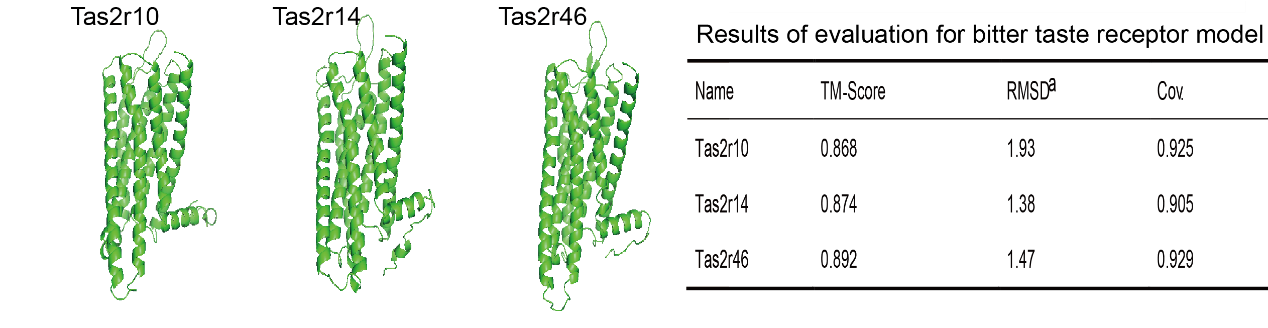


Fig.S.5 Bitter receptor homologous model. The bitter receptor homologous models of Tas2r10, Tas2r14, Tas2r46, and the evaluation results.
